# Improving water retention capacity of an aeolian sandy soil with feldspathic sandstone

**DOI:** 10.1038/s41598-019-51257-y

**Published:** 2019-10-11

**Authors:** Lu Zhang, Jichang Han

**Affiliations:** 1 Shaanxi Provincial Land Engineering Construction Group Co., Ltd, Xi’an, Shaanxi 710075 China; 2 Institute of Land Engineering and Technology, Shaanxi Provincial Land Engineering Construction Group Co., Ltd., Xi’an, Shaanxi 710075 China; 3 Key Laboratory of Degraded and Unused Land Consolidation Engineering, The Ministry of Natural Resources of the People’s Republic of China, Xi’an, Shaanxi 710075 China; 4 Shaanxi Provincial Land Consolidation Engineering Technology Research Center, Xi’an, Shaanxi 710075 China

**Keywords:** Agroecology, Environmental impact

## Abstract

The Mu Us sandy land in China’s Shaanxi Province faces a critical water shortage, with its aeolian sandy soil endangering the regional eco-environment. Here we investigated the effects of feldspathic sandstone on water retention in an aeolian sandy soil from the Mu Us sandy land. Feldspathic sandstone and aeolian sandy soil samples were mixed at different mass ratios of 0:1 (control), 1:5 (T1), 1:2 (T2), and 1:1 (T3). Soil-water characteristic curves were determined over low- to medium-suction (1–1000 kPa) and high-suction (1000–140 000 kPa) ranges, by centrifuge and water vapor equilibrium methods, respectively. Results showed that the addition of feldspathic sandstone modified the loose structure of the aeolian sandy soil mainly consisting of sand grains. The van Genuchten model described well the soil-water characteristic curves of all four experimental soils (*R*^2^-values > 0.97). Soil water content by treatment was ranked as T2 > T3 > T1 > control at the same low matric suction (1–5 kPa), but this shifted to T2 > T1 > T3 > control at the same medium- to high-suction (5–140 000 kPa). T2 soil had the largest saturated water content, with a relatively high water supply capacity. This soil (T2) also had the largest field capacity, total available water content, and permanent wilting coefficient, which were respectively 17.82%, 11.64%, and 23.11% higher than those of the control (*P*-values < 0.05). In conclusion, adding the feldspathic sandstone in an appropriate proportion (e.g., 33%) can considerably improve the water retention capacity of aeolian sandy soil in the study area.

## Introduction

Soil water shortage is often a key factor that limits crop production and agricultural development in desert. This factor constrains the selection and restoration of regional native vegetation, while it also determines soil productivity^1,2^. To solve the problem of soil water shortage in sandy lands, it is critical to improve soil infiltration and water retention capacity. Currently, efforts to implement sandy land improvement worldwide mainly involve sand blocking and sand control activities^3–5^. Great achievements are often realized through mechanical sand barriers^6,7^, chemical bonding^8–12^, and various biological sand-fixing techniques^13–15^. Yet, surprisingly little research has used local materials primarily for sand fixation to implement sandy land improvement via eco-environmental conservation.

The Mu Us sandy land encompasses those sandy land areas in the southeastern part of Ordos Plateau and along the Great Wall in northern Shaanxi Province, China^16^, bound by the geographical coordinates 37°30′–39°20′N and 107°20′–111°30′E. After the Yellow River channel was basically formed (100 000 to 10 000 years ago), a river paleolake formed a tableland via the alluvial and alluvial effects of flowing water; then, aeolian activity since the middle and late Quaternary created the present geomorphologic landscape of this sandy land^17^. In this region, feldspathic sandstone occurs across a wide area of ~16 700 km^2^. This soft rock is commonly found worldwide in deserts formed by marine, lacustrine, and continental deposits. So far, considerable reserves of feldspathic sandstone have been found at both the center and edge of sandy lands^18^.

In the Yellow River, feldspathic sandstone is the main source of coarse sand, sometimes called Earth’s “ecological cancer”. This material is characterized by easy weathering and poor water permeability, given its low diagenetic degree and structural strength along with its poor inter-particle cementation. Meanwhile, feldspathic sandstone also features high water retention and conservation capacity. Hence, a certain amount of water can be stored in a rock formation consisting of feldspathic sandstone, resulting in a rich water-bearing layer^19^. The abundant secondary clay minerals contained in feldspathic sandstone provide a rare yet ideal material for “water retention” in sandy lands^20–22^. These features point to a new idea for how to compensate for the poor water properties of aeolian sandy soil using feldspathic sandstone. In this context, investigating the benefits of feldspathic sandstone is of great significance to soil consolidation, ecological restoration, and agricultural development in sandy lands.

The soil-water characteristic curve (SWCC) can be used to describe the relationship between the water content and the matric suction of unsaturated soil^23^. Many mathematical models are used by soil physicists to fit the SWCC^24^. Those widely accepted and commonly applied include the Brooks-Corey model^25^, Gardner model^26,27^, Gardner-Russo model^28^, and van Genuchten model^29^. Because the Brooks-Corey, Gardner, and Gardner-Russo models are applicable to simulation of gradual change in soil water content, their applications are restricted by soil type. By contrast, the van Genuchten model has a wider range of application and is broadly used to the fit the data obtained from soils differing in their properties^30–36^.

In this study, the van Genuchten model was adopted to fit the SWCCs of an aeolian sandy soil modified by feldspathic sandstone at different mass ratios. Then soil water availability parameters were calculated to evaluate the effects of sandstone addition on water retention in the sandy soil. The optimal scheme for modifying aeolian sandy soil with feldspathic sandstone was discussed from the perspective of agricultural production. The results could inform and guide future sandy land improvement and production practices in the study region.

## Methods

### Study area

**Figure 1:**
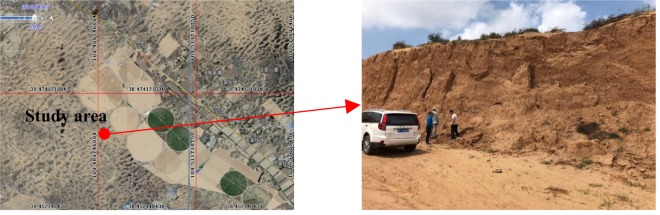
Remote sensing image of the study area generated using Google Earth Pro 7.1.8.3036 (https://earth.google.com/web/), and photo of feldspathic sandstone sampling site (photographed by L. Z.).

The study area is located in the Dajihan Village (109°28′E, 38°38′N), which is in the Yuyang District of Yulin, in northern Shaanxi Province, China (Fig. 1). This region has an altitude of 1000–1600 m. It features typical aeolian sandy grassland that is part of the Mu Us sandy land, where the annual average temperature is 6.0–8.5 °C. The coldest month is January with an average temperature of −9.5 to 12 °C, while July is the hottest with an average temperature of 22–24 °C. The annual accumulated temperature of ≥10 °C is 3000 °C. Annual precipitation is between 250 and 440 mm, concentrated primarily in summer and autumn (July–September account for 60–75% of total annual precipitation). However, the precipitation also shows considerable inter-annual variation, as it can be 2–4-fold higher in a wet than dry year. The maximum daily precipitation is 100–200 mm, while the air dryness is 1.0–2.5. Sand-blowing winds at speeds >5 m·s^−1^ occur 220 to 580 times per year, and the height of sand dunes does not exceed 10 m. In addition to aeolian sandy soil, both Jurassic sandstone and mudstone are also widely found in this region.

### Soil sampling and experimental treatments

We chose typical areas of aeolian sandy soil and feldspathic sandstone (purplish red) found in the study area, respectively. Five points, spaced 160 m apart, were selected in each 500-m × 400-m plot (*n* = 2 plots; Fig. 1). The sandy soil and sandstone samples were collected from a depth of 0–20 cm; their main physicochemical properties are shown in Table 1. For each material, samples from the five random points were mixed to form a composite sample per plot, then air-dried and passed through a 2-mm sieve. The sieved samples were mixed thoroughly at different mass ratios, feldspathic sandstone: aeolian sandy soil = 0:1 (control, CK), 1:5 (T1), 1:2 (T2), and 1:1 (T3).

**Table 1:** Physicochemical properties of the aeolian sandy soil and feldspathic sandstone used in this study.

Material	Aeolian sandy soil	Feldspathic sandstone (Fuchsia)
Bulk density (g·cm^−3^)	1.48 ± 0.0785	1.31 ± 0.0205
pH	7.6 ± 0.4784	8.2 ± 0.1700
Cation exchange capacity (cmol kg^−1^)	4.25 ± 0.1445	53.86 ± 1.2302
Organic matter (g·kg^−1^)	2.74 ± 0.3354	3.28 ± 0.4152
Total N (g·kg^−1^)	0.06 ± 0.0135	0.05 ± 0.0531
Total P (g·kg^−1^)	0.54 ± 0.0531	1.16 ± 0.0910
Total K (g·kg^−1^)	12.73 ± 1.1088	15.03 ± 0.7907

Values are the mean ± standard deviation (*n* = 3).

### Grain-size distribution analysis

Grain size of the experimental soils was analyzed by laser diffraction using the Mastersizer 2000 laser particle size analyzer (Malvern, Worcestershire, UK). The range of grain-size distribution was determined based on the Chinese system of grain size fractionation^23^, and soil mechanical composition was analyzed based on the USDA soil texture ternary diagram^23^.

### SWCC determination

The centrifuge method was used to determine the SWCC at low to medium matric suctions (up to 1000 kPa). With increasing speed of centrifugation (i.e., suction), the water and air in the soil were gradually expelled, leading to the decrease in soil volume and simultaneous increase in bulk density. Such changes could affect water retention in the soil and thus result in inaccurate measurements^37^. To address this issue, we immediately took the soil sample (contained in a centrifuge tube) from the centrifuge and weight it before changing the centrifugation speed each time. The shrinkage of the soil sample was measured using a vernier caliper in order to calculate the actual bulk density at each equilibrium point. The corresponding bulk density was then used to calculate the volumetric water content at each equilibrium point. The soil sample was immediately placed back into the centrifuge for the next step of centrifugation. Additionally, the water vapor equilibrium method was used for determining the SWCC at high matric suctions (up to 140,000 kPa)^38^. Finally, data from different suction ranges were fitted using the van Genuchten model.

(1) Low to medium matric suction (i.e., 1–1000 kPa): Each experimental soil sample was loaded into a 100-cm^3^ cutting ring at a fixed density of 1.35 g/cm^3^, with three replicates per treatment. After equilibrium was reached for water absorption and desorption (8–12 h), a dehydration test was performed using the CR21G III high-speed refrigerated centrifuge (Hitachi, Tokyo, Japan). The principle of the centrifuge method is expressed in Eq. (1):

1 $$pF=2\log \,N+\,\log \,h+\,\log ({r}_{1}\mbox{--}h/2)-4.95$$

where *pF* is the matric suction, namely the logarithm of the absolute value of centimeter water for soil matric potential *φ*, as shown in Eq. (2), *N* is the speed of centrifugation, $N=\sqrt{\frac{h}{1.18\times {10}^{-5}h({r}_{1}-\frac{h}{2})}}$, *r/S*; *h/2* is the half-height of soil sample, cm; and *r*_1_ is the radius of the centrifuge rotor, cm^37^. By using this method, a one-to-one correspondence can be established between water content and matric suction.

2 $${pF}=\,{\rm{lg}}(|\phi |\times {\rm{1020}})$$

where *φ* is the matric potential (bar).

The matric suction was set to 1, 3, 5, 7, 10, 30, 50, 70, 100, 200, 300, 500, 700, and 1000 kPa. The gravimetric water content of soil samples during the corresponding dehydration processes was measured by the centrifuge method and then converted to volumetric water content using the measured bulk density of the experimental soil^39,40^.

(2) High matric suction (i.e., 1000–140 000 kPa): Each experimental soil sample was placed inside a 20-mL weighing bottle and tested by the water vapor equilibrium method. The principle of the method is that the water vapor pressure varies with different concentrations of saline solutions^38^. Five saturated saline solutions (K_2_SO_4_, KCl, NaCl, Ca[NO_3_]_2_, and CaCl_2_·6H_2_O) were respectively placed into five desiccators to create microenvironments differing in their relative air humidity. The edge of the desiccator was sealed with Vaseline to ensure vacuum-like conditions. The weighing bottles were kept in such an environment to reach equilibrium of water vapor adsorption–desorption. All five desiccators were kept at a constant temperature of 20 °C, maintained by a water bath (i.e., continually filling water into a large metal box). Once the equilibrium was reached, the soil water content under different matric potential conditions was measured.

The relative air humidity and matric suction in the space of different saturated saline solutions at 20 °C were calculated using Eq. (3) ^38^.

3 $$pF=6.5+\,{\rm{lg}}(2-\,{\rm{lg}}(100RH))$$

where *pF* is the matric suction and *RH* is the relative humidity of the equilibrium air (%).

The matric suction *pF* was also calculated for the saturated saline solutions of K_2_SO_4_, KCl, NaCl, Ca(NO_3_)_2_, and CaCl_2_·6H_2_O using Eq. (1), i.e., 4.44, 5.32, 5.60, 5.91, and 6.16.

### Van genuchten model fitting

The van Genuchten model was applied to mathematically fit soil water content values measured over the two different suction ranges, alongside their matric suction values. Model fitting was implemented using the RETention Curve software (Salinity Laboratory, Agricultural Research Service, United States Department of Agriculture) with Eq. (4) ^29^, which gave the SWCC.

4 $${\theta }_{V}={\theta }_{r}+\frac{{\theta }_{s}-{\theta }_{r}}{{(1+{|\alpha {\phi }_{m}|}^{n})}^{m}}\,({\rm{where}},\,\phi  < 0,\,m=1-\frac{1}{n},\,{\theta }_{r}\le \theta \le {\theta }_{s})$$

where *θ*_r_ and *θ*_s_ are the residual (surplus) water content and saturated water content, respectively (%_V_); *θ*_r_ is the soil water content at $d(\theta )/d|{\phi }_{m}|$ = 0 (*φ*_m_ → ∞) (%_V_); *φ*_m_ is the soil matric potential (cm H_2_O); *α* and *n* are two shape parameters (empirically fitted parameters) of the curve, where *α* is the reciprocal of soil matric potential at the inflection point of air inflow on the curve when $d(\theta )/d|{\phi }_{m}|$ reaches its maximum (cm^−1^), and it is related to the volume of air inflow; *n* reflects the rate of the curve as it approaches the *y* axis, namely the curve’s slope; *m* is another shape parameter of the curve (*m* = 1–1/*n*); both *n* and *m* are related to soil structure as well as grain-size distribution and composition, reflecting the water retain capacity of the soil.

### Calculating water availability parameters

Field capacity is defined as the upper limit of soil water availability, and it is equivalent to the soil water content at a suction of 33 kPa. By contrast, the permanent wilting coefficient (i.e., unavailable water content) is the lower limit of soil water availability, and it is equivalent to the soil water content at a suction of 1500 kPa. When soil water content is below the permanent wilting coefficient, plant crops are difficult to grow and even wither to death. The water content between the field capacity and permanent wilting coefficient is considered to be available to plants for uptake, while the field capacity minus the permanent wilting coefficient conveys the maximum available water content, namely the total available water content in soil^41,42^.

Based on the difficulty of absorption, total available water content may be divided into quick available water (easily accessible water) and slow available water (hardly accessible water). The former is field capacity minus soil water content at a suction of 600 kPa, while the latter is soil water content at a suction of 600 kPa minus the permanent wilting coefficient^43^. The absolute content of soil available water is thus the ratio of quick available water to total available water content, and this parameter is typically used to characterize soil water availability for crops^44^.

### Data analysis

Data are expressed as the mean ± standard deviation (*n* = 3). Grain size data were analyzed and plotted using SigmaPlot 12.5 (Systat Software Inc., San Jose, USA). The SWCCs were drawn using Microsoft Excel 2016 (Microsoft Corp., Redmond, USA) and significant differences in soil water content between treatment groups were determined by paired-sample *t*-test. Water availability parameters were compared using one-way analysis of variance followed by Duncan’s new multiple range test. All statistical analyses were performed using SPSS Statistics 18.0 (SPSS Inc., Chicago, USA), with a *P*-value < 0.5 considered *a priori* to indicate statistical significance.

## Results

### Grain-size distribution

**Figure 2:**
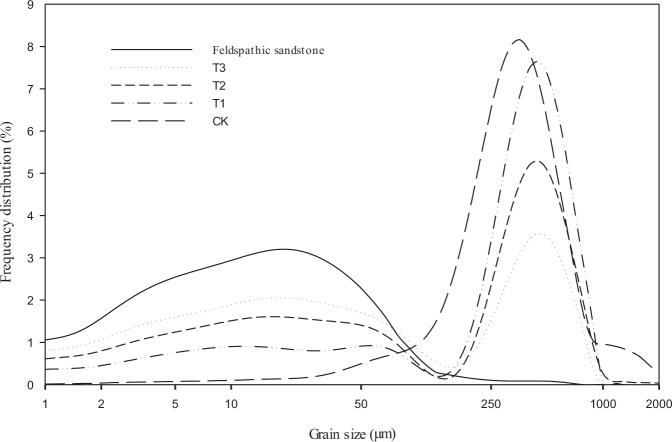
Grain-size frequency distribution curves of feldspathic sandstone, aeolian sandy soil (control, CK), and the mixture of the two materials (T1 = 1:5; T2 = 1:2; and T3 = 1:1).

Figure 2 shows the grain-size frequency distribution curves of feldspathic sandstone, aeolian sandy soil (CK), and the mixture of the two materials (T1, T2, and T3). The grain size of feldspathic sandstone had a wide range of distribution (0.001–0.25 mm), with no sharp peak on the frequency distribution curve (clay content = 14.26%). The grain size of aeolian sandy soil was mainly distributed between 0.05 and 1 mm. Generally, the sandy soil grains were coarse, with a narrow peak on the frequency distribution curve (clay content = 0.3%). After the addition of feldspathic sandstone to the aeolian sandy soil, the frequency distribution curves of T1 to T3 soils could be divided into two parts, with the grain size of 0.05 mm as the boundary. When grain size <0.05 mm, the content of fine-sized grains increased with increasing addition of feldspathic sandstone; however, when grain size >0.05 mm, the content of coarse-sized grains gradually decreased with increasing addition of feldspathic sandstone. T1, T2, and T3 soils had the clay content of 1.7%, 4.2%, and 5.1%, respectively.

### SWCC characteristics and fitting parameters

**Figure 3:**
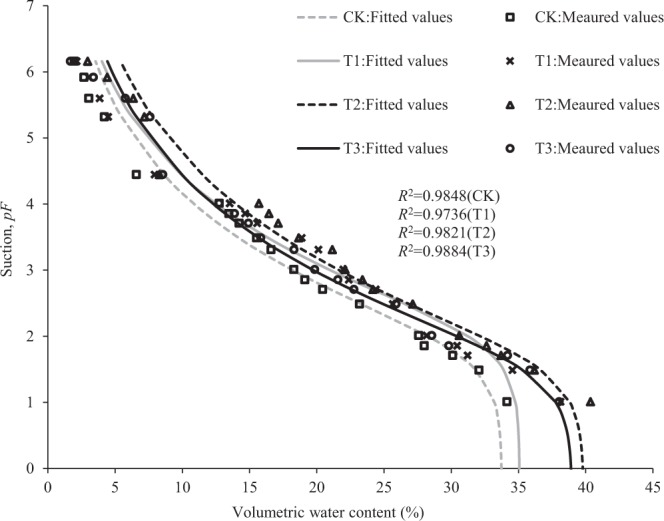
Soil-water characteristic curves of aeolian sandy soil modified by feldspathic sandstone at mass ratios (CK = 0:1, control; T1 = 1:5; T2 = 1:2; and T3 = 1:1).

The SWCCs for the experimental soils are shown in Fig. 3. The van Genuchten model described well the SWCCs of all four soils (*R*^2^-values > 0.97); in other words, the predicted values closely matched the observed ones. Comparing the four curves, the soil water content by treatment were ranked as T2 > T3 > T1 > CK (*P-*values < 0.05) at the same low matric suction (1–5 kPa), whereas this order changed to T2 > T1 > T3 > CK (*P-*values < 0.05) at the same medium to high matric suction (5–140 000 kPa). Table 2 gives the van Genuchten model parameters for the SWCCs of the experimental soils. The *θ*_r_ clearly differed among the treatments, with T2 generating the largest *θ*_s_ value. T2 soil also had lowest shape parameter (*m*) value for the SWCC.

**Table 2:** The van Genuchten model parameters of aeolian sandy soil (S) modified by feldspathic sandstone (F).

Mass ratio (F:S)	Saturated water content (*θ*_s_, %)	Residual water content (*θ*_r_, %)	Shape parameter (*α*, cm^−1^)	Shape parameter (*n*)	Shape parameter (*m*)
0:1 (Control, CK)	33.77 ± 0.5191b	4.5 ± 0.0816c	0.015 ± 0.0050b	1.226 ± 0.0155a	0.184 ± 0.0142a
1:5 (T1)	35.08 ± 0.6864b	5.7 ± 0.1700b	0.0089 ± 0.0031b	1.229 ± 0.0077a	0.186 ± 0.0079a
1:2 (T2)	39.85 ± 0.5717b	6.7 ± 0.1700a	0.024 ± 0.0031a	1.191 ± 0.0083a	0.160 ± 0.0045b
1:1 (T3)	38.99 ± 0.7736a	6.5 ± 0.1247a	0.027 ± 0.0019a	1.206 ± 0.0335a	0.171 ± 0.0087ab

Values are the mean ± standard deviation (*n* = 3). Means with different lowercase letters within a column are significantly different at *P* < 0.05.

### Soil water availability

**Figure 4:**
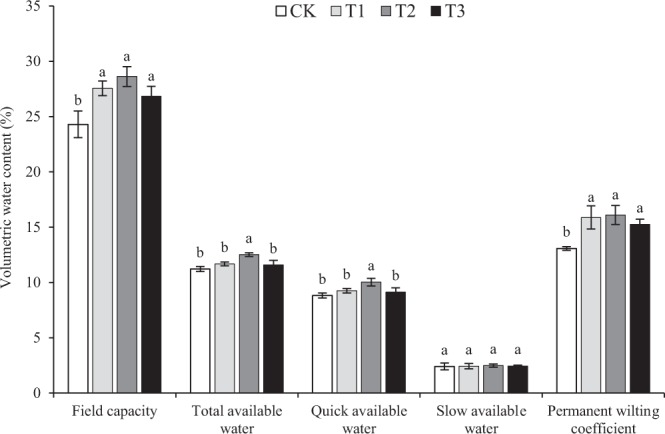
Water availability parameters of aeolian sandy soil modified by feldspathic sandstone and at different mass ratios (CK = 0:1, control; T1 = 1:5; T2 = 1:2; and T3 = 1:1). Values are the mean ± standard deviation (*n* = 3). For each parameter, different lowercase letters indicate a significant difference between means at *P* < 0.05.

The field capacity, permanent wilting coefficient, and available water content of the experimental soils are shown in Fig. 4. Differences among treatments were detected in soil water availability, i.e., T2 > T1 > T3 > CK. Both field capacity and permanent wilting coefficient increased significantly in the aeolian sandy soil after adding the feldspathic sandstone. The field capacity of T1, T2, and T3 soils was respectively 13.43% (*P* < 0.05), 17.82% (*P* < 0.05), and 10.44% higher relative to CK. In terms of total available water content, values of T1, T2, and T3 soils were respectively 4.18% (*P* < 0.05), 11.64% (*P* < 0.05), and 3.31% higher over those of CK. Among these parameters, slow available water showed the least changes across the different treatments. Further calculations revealed that the water availability of the experimental soils was ranked as follows: T2 > T1 > T3 > CK.

## Discussion

In the present study, we chose feldspathic sandstone from the Mu Us sandy land as a local sand-fixing material and mixed it with the aeolian sandy soil at different mass ratios. We found that adding the feldspathic sandstone in an appropriate proportion could reduce water percolation in the aeolian sandy soil while mitigating the hardening of the feldspathic sandstone *per se*^45^. Hence, by using this local sand-fixing material, our proposed strategy could improve the water retention capacity of aeolian sandy soil in the Mu Us sandy land in order to create favorable soil water conditions for crop growth. Our work therefore provides a baseline reference for sandy land management.

### Mechanisms of water retention in aeolian sandy soil added with feldspathic sandstone

Feldspathic sandstone is a soft rock composed of thick sandstone, sandy shale, and argillaceous sandstone^46^, which is rich in colloids. Studies have shown that soil colloidal particles contribute up to 80% of a soil’s surface charge^47,48^. Here our grain-size analysis revealed that the homogeneous grain-size composition of the aeolian sandy soil was modified after the addition of feldspathic sandstone. The grain-size composition of the modified soils (T1, T2, and T3) appeared to be a mixture of fine and coarse grains, with an expanded range of grain-size distribution. Considering the results obtained over the high- and low-suction ranges, the T2 treatment resulted in the best water condition at a given matric suction level. Hence, adding the feldspathic sandstone to an aeolian sandy soil at the mass ratio of 1:2 could improve soil water retention capacity and foster conditions suitable for enhanced soil water storage. According to the derived van Genuchten model parameters (*θ*_s_ and *θ*_r_), adding the feldspathic sandstone change the overall grain-size composition of aeolian sandy soil, thus affecting key soil attributes such as texture and structure. In particular, 33% of feldspathic sandstone (T2) in the aeolian sandy soil was most effective at forming an aggregate structure.

The T2 treatment also resulted in the lowest *n* and *m* values, which are shape parameters of the SWCC. This result indicates that the T2 soil had the lowest rate to approach the residual water content during dehydration, which also confirms the high water retention capacity of this soil. By contrast, the aggregation of regular aeolian sandy soil (CK) was extremely poor and its saturated water content was very low, with severe percolation of both water and nutrients. Corroborating this result was T2 having a relatively high water retention capacity, which may be related to the claying effect of feldspathic sandstone. Furthermore, it follows that this T2 treatment could provide better water support for crops. For engineering applications to the Mu Us sandy land, we recommend adding a small proportion (e.g., 1:2, by mass) of feldspathic sandstone to aeolian sandy soil. This strategy could ensure the highly efficient use of water by crops while saving on engineering costs.

### Practical implications of adding feldspathic sandstone into aeolian sandy soil for agricultural production

Generally, crops with a short growing period are suitable for cultivation in sandy soils, since these plants are at less risk of withering during later growth stages. Similarly, drought-tolerant (e.g., watermelon, sesame, and sorghum) and early-maturing crops also perform better in a sandy soil environment, and some tuberous crops (e.g., potatoes and sweet potatoes) produce higher yields in sandy soils^23^. The better growth of some crops in sandy soil has been verified by crop planting experiments, in which aeolian sandy soil was modified by feldspathic sandstone.

For example, in our land consolidation protect in the Dajihan Village (Yulin, Shaanxi Province), we mechanically mixed the feldspathic sandstone with aeolian sandy soil at the optimal mass ratio (i.e., feldspathic sandstone: aeolian sandy soil = 1:2) based on our experimental results. We then compacted the mixed soil to construct a tillage layer, which achieved the best water retention effects. Additionally, the yield of potato could reach 156.68 kg per ha when planted in a modified soil composed of the feldspathic sandstone and aeolian sandy soil at a 1:2 mass ratio (sandy loam)^49^. Moreover, vegetable crops often require the soil to have good drainage and loose texture conditions, such as that provided by sandy soil and loam^50^. Together with our results, we consider the modified soil composed of feldspathic sandstone and aeolian sandy soil at a 1:2 mass ratio (loamy sand) is also suitable for planting vegetables.

### Prospects for quantitative simulation of water characteristics in aeolian sandy soil added with feldspathic sandstone

From the perspective of analyzed water characteristics, we added a locally abundant material from the Mu Us sandy land (i.e., natural, non-polluting and colloid-rich feldspathic sandstone) to the aeolian sandy soil. This addition markedly compensated for the poor structure of aeolian sandy soil and improved its water retention. However, as our results demonstrated, the water retention capacity of modified soils did not necessarily increase with increasing ratio of the feldspathic sandstone added.

It is worth noting that the SWCCs of T1 and T3 soils intersect at *pF* = 4.44 and 1.85 (Fig. 3). When *pF* < 1.85 or *pF* > 4.44, the water retention capacity of T3 was higher than that of T1; however, when 1.85 < *pF* < 4.44, the water retention capacity of T1 was higher than that of T3. This contradictory result was due to the distinct water conservation effects of different mass ratio between the aeolian sandy soil and feldspathic sandstone. In the low-suction range of *pF* < 1.85, the change in soil water content was mainly caused by the variation in structural porosity. Within the high suction range of *pF* > 4.44, the change in soil water content was mainly due to the variation in water retention by molecules on the surface of soil grains^51^. Different ratios between the aeolian sand soil and feldspathic sandstone have varied effects for water conservation in the mixed soil; thus, more feldspathic sandstone is not necessarily better for water conservation in the sandy soil. Here we only simulated the effects of feldspathic sandstone which was less than or equal to aeolian sandy soil in mass. Further quantitative simulation is needed to verify whether the situation of water conservation differs when the amount of feldspathic sandstone added is higher than aeolian sandy soil.

## Conclusions

In this study, we modified an aeolian sandy soil by adding feldspathic sandstone at different mass ratios. This addition of feldspathic sandstone compensated for the poor water retention of the aeolian sandy soil, and the modified soils showed increased water retention capacity when compared with the control. Therefore, we propose that feldspathic sandstone can be used for soil improvement in the Mu Us sandy land, in order to improve water use efficiency and the ecological environment. However, the soil improvement gained by adding more feldspathic sandstone does increase with its proportion. By taking into account soil water availability, we recommend a mass ratio of 1:2 for adding feldspathic sandstone to the aeolian sandy soil. This strategy could strengthen the water retention capacity of aeolian sandy soil and meet the water demand of crop growth in the study area.
